# Modifiable risk factors that mediate the effect of insomnia on the risk of low back pain: a network mendelian randomization study

**DOI:** 10.1186/s41065-024-00341-z

**Published:** 2024-11-07

**Authors:** WeiSong Lu, YongQuan Wang, Yue An, MengZe Li, Sen Wang, Jie Lian, Hegui Xu

**Affiliations:** 1 Guizhou University of Traditional Chinese Medicine, Guiyang, Guizhou China; 2 Luzhou Hospital of Traditional Chinese Medicine, Luzhou, Sichuan China; 3 Department of Orthopaedics, The First Affiliated Hospital of Guizhou University of Traditional Chinese Medicine, Guiyang, Guizhou China

**Keywords:** Low back pain, Insomnia, Modifiable risk factors, Body mass index, Musculoskeletal disease, Mendelian randomization

## Abstract

**Background:**

Low back pain (LBP) and insomnia are common global health issues, but their relationship and potential mediators remain unclear. This study aimed to explore the impact of insomnia on LBP using mendelian randomization (MR) methods and analyze the mediating role of modifiable factors.

**Methods:**

Univariable MR (UVMR) analysis was employed to examine the causal relationship between insomnia and LBP, as well as the association between modifiable factors [smoking, alcohol consumption, body mass index (BMI), and type 2 diabetes (T2DM)] and LBP. Subsequently, multivariable MR (MVMR) analysis was conducted to explore the impact of insomnia on the mediation of LBP risk by modifiable factors.

**Results:**

In the UVMR analysis, insomnia [odds ratio (OR) = 2.95, 95%CI: 2.33–3.72)] and BMI (OR = 1.18, 95%CI: 1.02–1.37) were positively associated with the prevalence of LBP. The effects of smoking, alcohol consumption, and T2DM on LBP were not significant (*P* > 0.05). In the MVMR analysis, the proportion of mediation of BMI on the relationship between insomnia and LBP was 7.12%.

**Conclusion:**

This study revealed the causal relationship between insomnia and LBP using MR methods for the first time, and identified the mediating role of BMI. These findings offer new insights into understanding the relationship between insomnia and LBP, informing the prevention and treatment of these two health issues.

**Supplementary Information:**

The online version contains supplementary material available at 10.1186/s41065-024-00341-z.

## Introduction

Low back pain (LBP) is a leading cause of disability worldwide, significantly burdening health systems and affecting the quality of life and social function of hundreds of millions of people [1–3]. The complex etiology includes various factors such as soft tissue injury, intervertebral disc herniation, and neurovascular structural damage [4]. It is closely related to factors such as obesity, physical labor, and psychological issues [5, 6]. However, 90% of patients fail to identify the fundamental cause [7]. Thus, it is crucial to identify the risk factors associated with LBP to guide public health strategies, reduce treatment costs, and enhance overall quality of life.

Insomnia, as another common health problem, its impact on individuals and society is equally significant. The definition of insomnia includes symptoms such as difficulty falling asleep, difficulty maintaining sleep, or early awakening [8], which may lead to decreased cognitive function, mood swings, and impaired social functioning [9, 10]. According to statistics, over 30% of the global population will experience one or more symptoms of insomnia, and this trend is on the rise [11]. Studies have shown a close relationship between insomnia and chronic pain, especially LBP [12–14]. Additionally, numerous observational studies have confirmed that modifiable risk factors such as smoking, alcohol consumption, body mass index (BMI), and type 2 diabetes (T2DM) are closely associated with insomnia and LBP [15, 16]. However, considering residual confounding factors and reverse causality, whether they may serve as mediators remains challenging to ascertain.

Mendelian randomization (MR) is a method that uses genetic variation as instrumental variables (IVs) to infer causal relationships between exposure and outcome [17]. Through the stability and random segregation of genetic variation, MR minimizes the influence of confounding and reverse causality, being regarded as a “natural” randomized controlled trial [18].

This study aims to explore the correlation between insomnia and LBP, and further analyze the mediating role of modifiable risk factors between them. We hope to provide new insights and strategies for the prevention and treatment of insomnia and LBP through this study, thereby improving patients’ quality of life and social function.

## Methods

### Study design

We used MR method to study the influence of insomnia on LBP and the mediating effect of modifiable risk factors. Initially, we employed the univariable mendelian randomization (UVMR) method to examine the causal relationship between insomnia and LBP, as well as the association between modifiable risk factors and LBP. Then, we identified modifiable risk factors causally related to LBP and conducted UVMR analysis with insomnia. Finally, potential intermediate factors were explored through multivariable Mendelian randomization (MVMR) analysis, and the mediating effects of these intermediate factors on insomnia and LBP were evaluated. When reporting the MR study, this study strictly followed the guidance of the STROBE-MR guidelines [19].

### Data sources

Genetic associations related to insomnia are derived from the UK Biobank and 23andMe, with this analysis incorporating a large sample of 1,207,228 individuals of European ancestry, making it one of the largest genome-wide association studies (GWAS) on insomnia to date [20]. Genetic variation data for LBP are sourced from the FinnGen Consortium (https://www.finngen.fi), including 13,178 cases and 164,682 European ancestry controls. The diagnosis of these cases is based on the International Classification of Diseases criteria by the World Health Organization. Genetic instruments for smoking and alcohol consumption are derived from statistical data of large-scale publicly available genome-wide association meta-analyses [21]. Additionally, data related to BMI are sourced from a global survey of physical health involving 806,834 individuals of European ancestry. BMI values were obtained through measurement of standing height and weight during the initial assessment center visit [22]. Data on T2DM, including 74,124 cases and 824,006 European ancestry controls, are sourced from a study by Anubha Mahajan et al. [23]. Table 1 summarizes the GWAS information studied.

**Table 1:** Summarized information for GWASs included

Trials	Author or Consortium	Year	Sample size	Population	PMID
Insomnia	Watanabe et al.	2022	1,207,228	European	35,835,914
Smoking	Liu et al.	2019	1,232,091	European	30,643,251
Alcohol consumption	Liu et al.	2019	941,280	European	30,643,251
BMI	Pulit et al.	2019	806,834	European	30,239,722
T2DM	Mahajan et al.	2018	898,130	European	30,297,969
LBP	FinnGen	2021	177,860	European	NA

GWAS, Genome-wide association studies; BMI, body mass index; T2DM, type 2 diabetes mellitus; LBP, Low back pain

### Instrumental variable selection

Specific criteria were implemented for IV selection in MR analysis. These criteria include: (1) establishing significant genome-wide associations between IV and exposure (*P* < 5.00 × 10^− 8^), (2) clustering within a 10,000 kb window and minimizing linkage disequilibrium (R^2^ < 0.01), and (3) setting the minimum minor allele frequency (MAF) threshold to 0.01. We used the F-statistic to assess the strength of IVs, considering values greater than 10 as indicating a low probability of weak instrument bias [24]. The formula for calculating the F-statistic is as follows: F = ((N-2) * R^2^ / (1-R^2^)), where R^2^ signifies the extent to which the single nucleotide polymorphism (SNP) explains the exposure, and N denotes the sample size [24]. The calculation formula for R^2^ is as follows: R^2^ = 2 × MAF × (1 - MAF) × β^2^, where MAF denotes the minor allele frequency, and β represents the effect size of the exposure [25].

### Statistical Analysis

#### Univariate MR analysis

In our study, we employed the inverse variance weighted (IVW) method as the primary method for univariable analysis. This technique utilizes a random-effects meta-analysis approach to combine results obtained from individual SNPs. To ensure consistency in the causal direction, we also conducted additional analyses using other methods, including MR Egger, weighted median, weighted mode, and simple mode, which should align directionally with IVW results to ensure the reliability of the findings. Additionally, we used the MR-Egger intercept to assess potential pleiotropy and Cochrane Q statistic to assess heterogeneity.

#### Multivariable MR analysis

MVMR analysis was utilized to explore the causal effect of insomnia on LBP risk mediated by modifiable risk factors. Initially, in UVMR analysis, we computed the overall causal effect of insomnia on LBP. Subsequently, we multiplied the estimates of the effect of insomnia on modifiable risk factors from UVMR analysis by the estimates of the effect of modifiable risk factors on LBP from MVMR analysis to obtain the indirect effect. Finally, the proportion mediated by modifiable risk factors was obtained by dividing the indirect effect by the total causal effect.

All statistical analyses in this study were conducted using the “TwoSampleMR” package in R software (version 4.1.0). Our study analyzed only publicly available summary-level statistical data obtained with approval from respective research ethics committees, thus institutional review board approval was not required.

## Results

### Instrumental variables

Detailed information on SNPs for each phenotype was obtained from GWAS and can be found in Supplementary Tables 1–5. A total of 427 SNPs were selected as IVs for insomnia, with an F-statistic of 19.96. For smoking, alcohol consumption, BMI, and T2DM, we extracted 19 SNPs (F = 47.26), 33 SNPs (F = 22.90), 59 SNPs (F = 29.20), and 123 SNPs (F = 31.20), respectively, ensuring there were no weak IVs.

### Effects of insomnia and modifiable risk factors on LBP

**Fig. 1:**
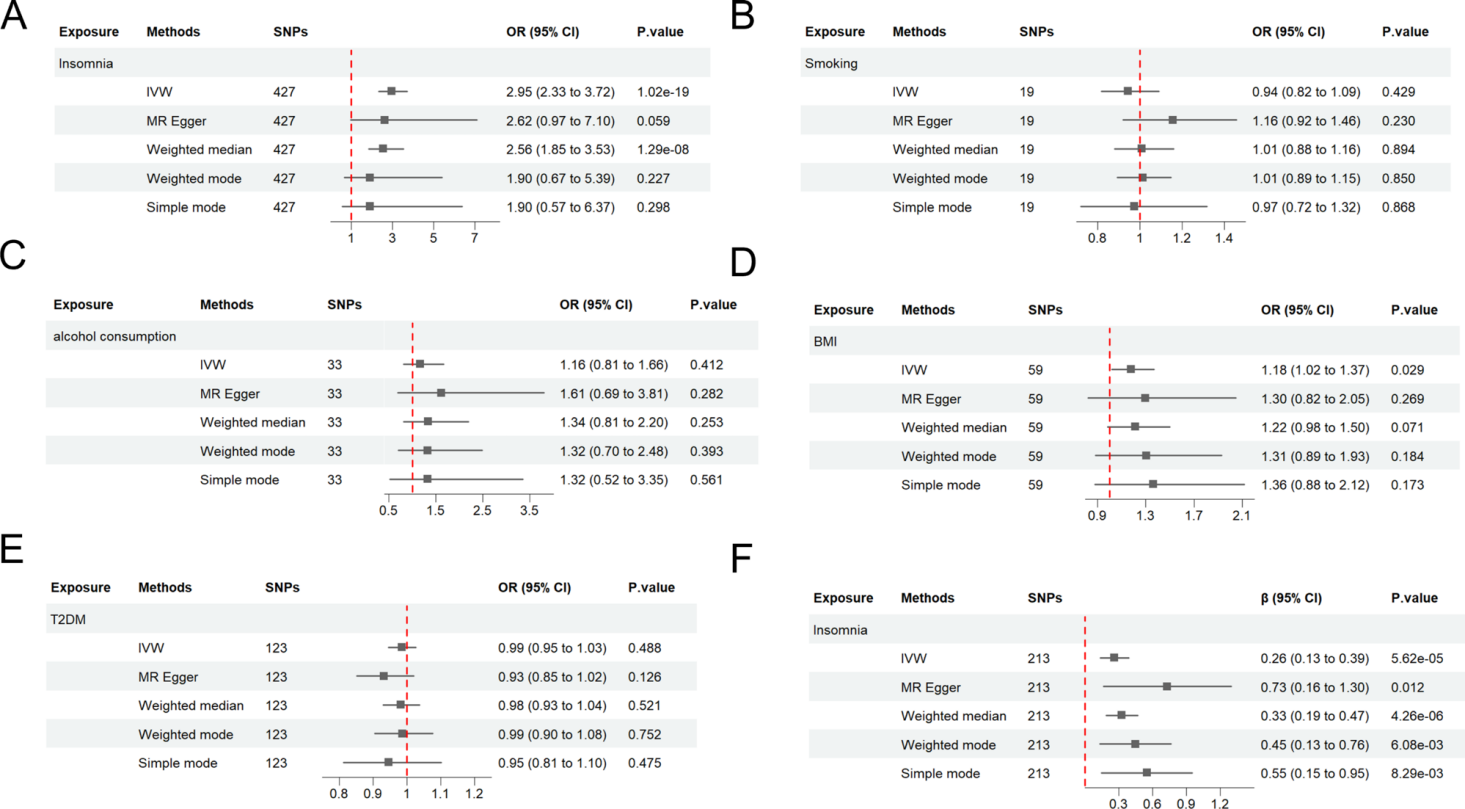
Univariate Mendelian randomization analysis. (**A**) The effects of insomnia on LBP. (**B**) The effects of smoking on LBP. (**C**) The effects of alcohol consumption on LBP; (**D**) The effects of BMI on LBP; (**E**) The effects of T2DM on LBP; (**F**) The effects of insomnia on BMI. LBP, low back pain; BMI, body mass index; T2DM, type 2 diabetes mellitus; SNP, single nucleotide polymorphism; OR, odds ratio; IVW, inverse variance weighting

In UVMR analysis, insomnia was associated with a risk of LBP, with an odds ratio (OR) of 2.95 (95%CI: 2.33 to 3.72). For the four potential mediators analyzed, smoking (OR = 0.94; 95%CI: 0.82 to 1.09), alcohol consumption (OR = 1.16; 95%CI: 0.81 to 1.66), and T2DM (OR = 0.99; 95%CI: 0.95 to 1.03) were unrelated to LBP. The association between BMI and LBP risk was 1.18 (95%CI: 1.02 to 1.37). The effect of insomnia on BMI was (β = 0.26, 95%CI: 0.13 to 0.39). These findings were illustrated in Fig. 1.

### Mediating effect

**Fig. 2:**
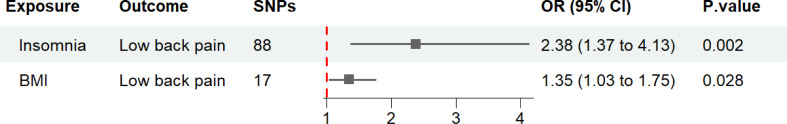
Multivariate Mendelian randomization analysis. The OR was derived from the method of inverse variance weighting. BMI, body mass index; SNP, single nucleotide polymorphism; OR, odds ratio

In MVMR analysis, smoking, alcohol consumption, and T2DM had no effect on LBP and were therefore excluded from subsequent MVMR analysis. After adjusting for BMI, the effect of insomnia on LBP risk was (OR = 2.38, 95%CI: 1.37–4.13), and the causal effect of BMI on LBP was (OR = 1.35, 95%CI: 1.03–1.75) (see Fig. 2).

**Fig. 3:**
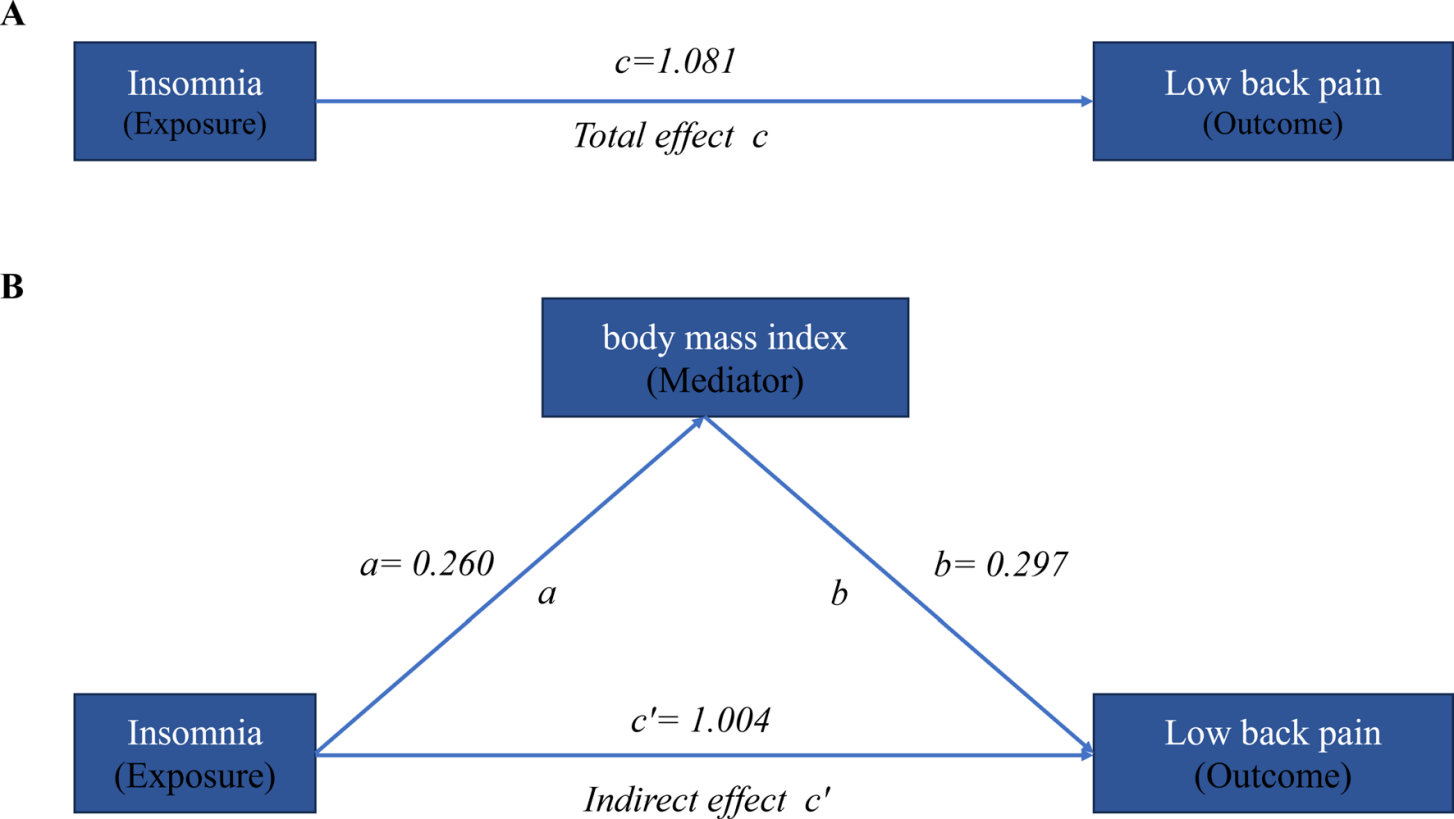
Total effect of insomnia on LBP (**A**) and mediating effects model for BMI (**B**) The efficiency values were derived from the method of inverse variance weighting. (**A**) The total effect of insomnia on LBP, c, was derived using univariable MR (i.e. genetically predicted insomnia as exposure and LBP as outcome). (**B**) The total effect was decomposed into: (i) indirect effect using a two-step approach (where a is the total effect of insomnia on BMI, and b is the effect of BMI on LBP adjusting for insomnia) and the product method (a × b) and (ii) direct effect (c′ = c – a × b). LBP, low back pain; BMI, body mass index

In the UVMR analysis, the effect of insomnia on LBP (c, as shown in Fig. 3 was 1.081 (95% CI: 0.848 to 1.314). The effect of insomnia on BMI (a, as shown in Fig. 3) was 0.260 (95% CI: 0.134 to 0.387). In the multivariable MR analysis, the effect of BMI on LBP (b, as shown in Fig. 3) was 0.297 (95% CI: 0.031 to 0.561). The proportion mediated by BMI was 7.12% (95% CI: 0.51%, 16.52%) (see Fig. 3) .

### Sensitivity analysis

In the MR analysis, significant heterogeneity was observed among the chosen IVs (*P* < 0.05). Consequently, the IVW random-effects model was employed for all MR analyses (see Table 2). Furthermore, MR-Egger intercept analyses found no evidence of potential horizontal pleiotropy (*P* > 0.05), indicating that the selected IVs did not significantly influence the outcome through pathways unrelated to the exposure (see Table 2).

**Table 2:** Heterogeneity and pleiotropy assessment

Exposure	Outcome	Cochran’s Q (IVW)	*P* for Cochran’s Q (IVW)	*P* for MR–Egger regression intercept	R2	F
Insomnia	LBP	564.545	< 0.001	0.810	1.65E-05	19.962
Smoking	LBP	35.636	0.008	0.482	3.84E-05	47.261
alcohol consumption	LBP	36.902	0.253	0.413	2.43E-05	22.901
BMI	LBP	60.198	0.396	0.667	3.62E-05	29.197
T2DM	LBP	173.180	0.002	0.172	3.47E-05	31.195
Insomnia	BMI	556.234	< 0.001	0.097	1.69E-05	20.401

IVW: inverse variance weighted; BMI, body mass index; T2DM, type 2 diabetes; LBP, Low back pain

## Discussion

In the MR analysis, we observed a positive correlation between insomnia and the prevalence of LBP. BMI was considered to be a key mediator in the causal relationship between insomnia and LBP. We did not observe smoking, alcohol consumption, and T2DM mediating the relationship between insomnia and LBP. To the best of our knowledge, this was the first MR study to investigate the causal relationship between insomnia and LBP and examine the role of modifiable risk factors (smoking, alcohol consumption, BMI, and T2DM).

Our findings are consistent with previous research, which has established a link between insomnia and an increased risk of LBP. For instance, a prospective study demonstrated that insomnia significantly elevates the risk of developing LBP, even after accounting for various confounding factors such as socioeconomic status, lifestyle behaviors, and anthropometric measures [26]. Similarly, a cross-sectional study revealed that individuals with insomnia were twice as likely to experience chronic LBP compared to those without sleep disturbances [27]. Furthermore, a Mendelian randomization study supported these results, suggesting that insomnia, rather than daytime sleepiness, plays a direct role in increasing the susceptibility to LBP [7]. Collectively, these studies underline the importance of addressing sleep disorders as a modifiable factor in the prevention and management of LBP, reinforcing the potential benefits of early intervention in individuals with insomnia. Further research has revealed that insomnia may affect back health through changes in inflammation gene expression mediated by epigenetics [28]. This inflammatory process may accelerate aging, leading to more severe lumbar pain. Additionally, insomnia is also associated with changes in pain modulation and central sensitization, which may further exacerbate symptoms of LBP and functional impairment [29]. These research findings provide a deeper understanding, indicating a complex and close association between insomnia and LBP, involving multiple aspects such as inflammation, aging, and neurobiology.

Mediation analysis showed that BMI was an intermediate variable in the causal pathway from insomnia to LBP. A study in Spain targeting adults aged 60 and above found that participants sleeping less than 5 h per day were more likely to develop obesity compared to those sleeping over 5 h [30]. In a study in Mexico, adults aged 50 and above showed an association between insomnia and obesity, especially among non-obese individuals [31]. Furthermore, a survey in Australia indicated a correlation between short sleep duration and obesity among individuals aged 55 to 64 [32]. These study findings highlight the close relationship between insomnia and obesity. Its possible mechanism involves the dysregulation of neuroendocrine control of appetite in insomnia, associated with decreased satiety factor leptin and increased hunger-promoting hormone ghrelin [33]. Additionally, insomnia may lead to metabolic disorders, affecting glucose metabolism and insulin sensitivity, thereby increasing the risk of obesity [34]. Therefore, insomnia may promote obesity by affecting metabolism and hormone levels. In addition, numerous previous studies have also confirmed that obesity increases the risk of developing LBP [35, 36]. Because obesity increases the load on the spine and joints, it may lead to changes in spinal structure, resulting in the occurrence of LBP [37]. Obesity also leads to an increase in the body’s inflammatory levels, as excessive accumulation of adipose tissue releases a series of inflammatory mediators, which may affect the regulation of the nervous system, increasing pain perception and the occurrence of low back pain [38]. In summary, insomnia may promote obesity by affecting metabolism and hormone levels, while obesity may cause LBP through mechanisms such as metabolic disorders, inflammatory status, and changes in spinal structure, thus constituting the complex association among insomnia, obesity, and LBP.

Despite our findings suggested that insomnia may be associated with other modifiable risk factors that increase the risk of LBP, our analysis suggested that BMI explained only a small proportion of the causal relationship. In sensitivity analyses, smoking, alcohol consumption, and T2DM seem to have no meaningful causal impact on the risk of LBP. These null results contrast with findings from many observational studies demonstrating associations between these factors and LBP risk, which may reflect unmeasured confounding, measurement errors, or other biases [39–41]. Certainly, null results may also stem from challenges in accurately measuring these exposures in GWASs. Hence, further research is warranted to comprehensively understand the relationship between insomnia, BMI, and other modifiable risk factors and LBP.

One major advantage of our study is the first-time use of two-step MR to explore the mediating effects of modifiable risk factors (smoking, alcohol consumption, BMI, and T2DM) in the causal relationship between insomnia and LBP”. This method overcomes inherent biases in traditional epidemiology such as residual confounding, reverse causation, and measurement errors. However, our study has some limitations. Firstly, while we attempt to explain the relationship between insomnia, BMI, and other modifiable factors with LBP, there may be complex interactions and nonlinear relationships among these factors, increasing the complexity of result interpretation. Secondly, despite applying stringent criteria in selecting IVs, there may still be uncertainty in instrument selection in MR studies, including potential IVs that were not considered or did not fully represent the relationship between the target exposures and outcomes. Thirdly, although physical activity and sedentary behavior are important factors, they were not included as mediators due to the lack of robust genetic instruments in the available GWAS data. Additionally, the GWAS data did not specify whether LBP was acute, sub-acute, or chronic, which is a notable limitation in interpreting the underlying pathophysiological mechanisms. Finally, the genetic association data used in this study are from databases such as the UK Biobank, 23andMe, and the FinnGen Consortium, which may suffer from issues of representativeness, particularly regarding insufficient data for specific populations or regions, thus limiting the generalizability and applicability of the findings.

## Conclusion

In conclusion, this study revealed the causal relationship between insomnia and LBP, identifying BMI as a small but significant mediator. This finding underscores the importance of insomnia and obesity management in preventing LBP. Future research should further explore other potential mediators and conduct clinical trials of intervention measures to improve patients’ quality of life. Clinicians managing individuals with low back pain should emphasize the importance of adequate sleep and maintaining a healthy BMI to reduce risk and effectively manage low back pain.

## Electronic Supplementary Material

Below is the link to the electronic supplementary material.

Supplementary Material 1

## Data Availability

All the Mendelian randomization study files are available from GWAS. (URL https://gwas.mrcieu.ac.uk).

## Abbreviations

LBP: low back pain
MR: mendelian randomization
UVMR: univariate mendelian randomization
MVMR: multivariable mendelian randomization
BMI: body mass index
T2DM: type 2 diabetes
IVs: instrumental variables
GWAS: Genome-wide association studies
MAF: minimum allele frequency
IVW: inverse variance weighting
SNP: single nucleotide polymorphism
OR: odds ratio
CI: confidence interval
β: regression coefficient
SE: standard error
